# Obesity-Induced Upregulation of ZBTB7A Promotes Lipid Accumulation through SREBP1

**DOI:** 10.1155/2020/4087928

**Published:** 2020-01-07

**Authors:** Jing-ping Zhou, Yan-dan Ren, Qin-yu Xu, Yang Song, Fei Zhou, Mei-Ya Chen, Jing-jing Liu, Li-Gang Chen, Jin-Shui Pan

**Affiliations:** Department of Gastroenterology, Zhongshan Hospital Affiliated to Xiamen University, Xiamen 361000, China

## Abstract

**Objective:**

Nonalcoholic fatty liver disease (NAFLD) is among the most common chronic liver diseases. However, the pathogenesis of NAFLD is not still unclear. This study aims at evaluating the role of zinc finger and BTB domain-containing 7A (ZBTB7A) in NAFLD.

**Methods:**

Western blotting, real-time reverse transcription PCR (RT-PCR), and immunohistochemistry were submitted to evaluate the level of ZBTB7A in the high fatty diet- (HFD-) induced NAFLD mouse model. *In vitro*, the expression of ZBTB7A was assessed in oleic acid- (OA-) induced HepG2 cells with western blotting and RT-PCR. The luciferase reporter assay was used to estimate the effect of ZBTB7A on the SREBP1 and NF-*κ*B, and the ChIP assay was subjected to evaluate the direct binding to the SREBP1 promoter. Oil Red staining was used to detect lipid accumulation, and the ELISA was used to verify the levels of TG, T-CHO, and MDA. ZBTB7A was knocked down with siRNA, and RT-PCR was performed to analyze the lipogenesis-, fatty acid transporter-, and oxidation metabolism-related genes expression. The levels of ZBTB7A in primary hepatocyte, Kupffer, and hepatic stellate cells (HSCs) were tested by RT-PCR.

**Results:**

The upregulation of ZBTB7A expression was assessed in NAFLD mice, and ZBTB7A expression was positively correlated with TNF*α*, IL-6, TG, T-CHO, and MDA. ZBTB7A was highly expressed in the hepatocytes. *In vitro*, OA-induced ZBTB7A expression and ZBTB7A expression were closely associated with SREBP1c. ZBTB7A could activate the promoter activity of SREBP1 and activate NF-*κ*B activity. Interestingly, the direct binding of ZBTB7A in the SREBP1 promoter was acquired in HepG2 cells. Inhibition of ZBTB7A expression could attenuate OA-induced lipid accumulation, inhibit the expression of the lipogenesis-related genes and fatty acid transporter genes, and promote the expression of oxidation metabolism-related genes.

**Conclusion:**

ZBTB7A plays a significant role in the development process of NAFLD, and obesity-induced upregulation of ZBTB7A promotes lipid accumulation through activation of SREBP1 and NF-*κ*B. ZBTB7A may be a potential novel target for the therapy of NAFLD.

## 1. Introduction

Nonalcoholic fatty liver disease is a common disorder of metabolism; it is a leading cause of liver fibrosis, type 2 diabetes, and some metabolic syndromes [1, 2]. Also, fibrosis is an irreversible pathological process, which could further progress into cirrhosis and hepatocellular carcinoma. Patients with NAFLD show liver steatosis, with or without hepatitis and fibrosis [3–5]. Built on the presence or absence of hepatitis, NAFLD is subdivided into nonalcoholic steatohepatitis (NASH) and nonalcoholic fatty liver (NAFL). In clinical practice, there is no histological difference between NAFLD and alcoholic fatty liver disease (AFLD), and liver biopsy is also universal in the diagnosis of NAFLD [6, 7]. Previous studies have demonstrated that the pathogenesis of NAFLD is very complicated and that many factors are involved in the progress of the disease. Some researchers showed that insulin resistance is universal in NAFLD patients [8–10]. Also, oxidative stress and lipid peroxidation are the outcomes of progression from fatty liver to steatohepatitis [11]. Furthermore, the occurrence of fibrosis was also an important risk factor in the progression of NAFLD [12]. However, there is no effective proposal for the therapy of NAFLD. The treatment is mainly focused on the control of NASH and the protection strategy of liver function [13]. Thus, the discovery of a potent therapeutic target for NAFLD is very urgent.

Zinc finger and BTB domain-containing 7A (ZBTB7A), also named as Pokemon, is a transcription factor widely expressed in humans [14]. According to HPA RNA-seq data, ZBTB7A is highly expressed in the stomach, colon, and skin and less expressed in the liver, spleen, and lymph node. Furthermore, many reports demonstrated that ZBTB7A was overexpressed in some cancers, acting as tumor promoter involved in the progression of the disease [15–18]. ZBTB7A was reported overexpressed in non-small-cell lung cancer and contributed to the inactivation of p53 [15]. Besides, ZBTB7A was associated with the chemoresistance of osteosarcoma [19]. In contrast, ZBTB7A was also reported as a tumor suppressor in prostate cancer, melanoma, triple-negative breast cancer, and gastric cancer [15, 20, 21]. Also, ZBTB7A was recognized as an insulin-responsive transcription factor, which participated in the regulation of hepatocyte glucose production [22]. However, the role of ZBTB7A in the NAFLD has not been reported yet; then in this study, ZBTB7A was significantly upregulated in the HFD-induced mouse model of NAFLD and correlated with proinflammatory factors, TG, cholesterol, and MDA levels. In addition, ZBTB7A could enhance the expression of SREBP1 through activation of the promoter activity. ZBTB7A also activated the activity of NF-*κ*B. Also, knockdown of ZBTB7A attenuated OA-induced lipid accumulation. Therefore, our study demonstrates that ZBTB7A might be a potential target for NAFLD.

## 2. Materials and Methods

### 2.1. Materials

Anti-ZBTB7A antibody (#20565s) was purchased from Cell Signaling Technology; anti-GAPDH antibody (Cat. 60004-1), mouse TNF*α* ELISA kit, and mouse IL-6 ELISA kits were purchased from Proteintech Inc.; anti-SREBP1c antibody was obtained from Santa Cruz Biotechnology. Oleic acid (CAS. 112-80-1, Lot. O1383) reagent was obtained from Sigma-Aldrich. TRIzol RNA extraction reagent (Cat. 10606ES60), 1st strand cDNA synthesis kit with gDNA digester (Cat. 11123ES10), qPCR SYBR Green Master Mix with high ROX (Cat. 11203ES03), immunohistochemistry kit for rabbit primary antibody (Cat. 36312ES75), and hematoxylin and eosin staining kit (Cat. 60524ES60) were purchased from Yeasen Biotech Co., Ltd; Oil Red staining kit (Cas. E607319) and the primer were obtained from Sangon Biotech (Shanghai) Co., Ltd. TG (Cas. A110-1-1), T-CHO (Cas. A111-2-1), and MDA (Cas. A003-1-1) were purchased from Nanjing Jiancheng Bioengineering Institute.

### 2.2. Animal Models

C57BL/6 mice were obtained from the SILAC animal Co. Ltd. Mice were housed under standard conditions with free access to food and water. All experimental procedures were approved by the Animal Welfare Committee of Research Organization, Xiamen University.

### 2.3. Western Blotting Analysis

Proteins were extracted from the liver tissues or cell lines in the lysis buffer consisting of 50 mM Tris-HCl, pH 8.0, 50 mM KCl, 5 mM DTT, 1 mM EDTA, 0.1% SDS, 0.5% Triton X-100, and protease inhibitor cocktail tablets. The extracted proteins were separated by polyacrylamide SDS gel and electrophoretically transferred onto polyvinylidene fluoride membranes. The membranes were probed with the indicated antibodies overnight at 4°C. Antibodies used in western blot were ZBTB7A (Proteintech Co. Ltd, 1 : 500 dilutions), GAPDH (CST, 1 : 1000 dilution), and SREBP1c (Santa Cruz, 1 : 500 dilution). And PVDF membranes were subsequently incubated with a horseradish peroxidase-coupled secondary antibody. Detection was carried out using a GE chemiluminescent substrate system.

### 2.4. Oil Red Staining

Liver tissue from NAFLD mice was fixed in formalin for at least 24 h and then embedded in paraffin. Tissue sections were stained with hematoxylin-eosin (H&E). HepG2 and constructed stable knocking down of ZBTB7A cells were fixed in 10% formalin for 30 min and stained in Oil Red O. Lipid droplets in cells were eluted with isopropanol, and the absorbance of the solution was monitored using an ELISA reader at a wavelength of 450 nm.

### 2.5. Immunohistochemistry (IHC) Assay

Mouse liver tissue sections were immune-stained with anti-ZBTB7A (1 : 100) antibody. Slides were counterstained with hematoxylin. For cell microscopy, HepG2 and stable knocking down of ZBTB7A cell lines were stained with Oil Red O reagent according to the manufacturer. Cells were further costained with hematoxylin to visualize nuclei.

### 2.6. Enzyme-Linked Immunosorbent Assay (ELISA)

Blood was taken from the eyes of the mice and then kept at room temperature for 2 h. After that, the fresh blood was centrifuged at 1000 g for 2 min. The supernatant was collected for analysis to TNF*α*, IL-6, TG, and T-CHO, and MDA biochemical indicator was analyzed according to the manufacturers.

### 2.7. Quantitative Real-Time (qRT-PCR) Assay

Total RNA was extracted using TRIzol, and the first cDNA was synthesized using First Strand cDNA Synthesis Kits (Yeasen). qRT-PCR was performed using High Rox SYBR® Green PCR Master Mix (Yeasen). Normalization was performed with GAPDH.

### 2.8. The Chromatin Immunoprecipitation (ChIP) Assay

A previous study showed that ZBTB7A could bind to GC-rich sequence [23], and the promoter sequence of SREBP1 was assessed in GeneCopoeia (Germantown, MD, USA). Putative binding sites of SREBP1 were analyzed in the AliBaba 2.1 portal. And the GC-rich sites were included to examine the binding activity of ZBTB7A on SREBP1 promoter. The primer sequence for potential binding sites is as follows: forward, 5′-GCC ATT GTG CGC GAG GCT GGA-3′; reverse, 5′-CCT CGG AAA CTG GGT TCC CC-3′. The HepG2 cells were transfected with vector and ZBTB7A-overexpressing plasmid for 24 h, and the ChIP assay was guided by the instruction of SimpleChIP Plus Sonication Chromatin IP Kit (Cell Signaling Technology, Beverly, MA, USA). The anti-ZBTB7A antibody (13E9, sc-33683) for ChIP was obtained from Santa Cruz Biotechnology (1 : 100 dilution). And the obtained DNA was subjected to qRT-PCR assay to evaluate the DNA level.

### 2.9. Statistical Analysis

All the results in this study are described as means ± SD. Statistical analysis was performed with Student′s *t*-test with two-tailed. And the correlation between the two groups was performed with the Pearson correlation coefficient. The statistical analysis was performed with GraphPad Prism 7. *P* value < 0.05 was regarded as the statistical difference.

## 3. Results

### 3.1. ZBTB7A Was Highly Expressed in OA-Induced NAFLD Mouse Model

**Figure 1:**
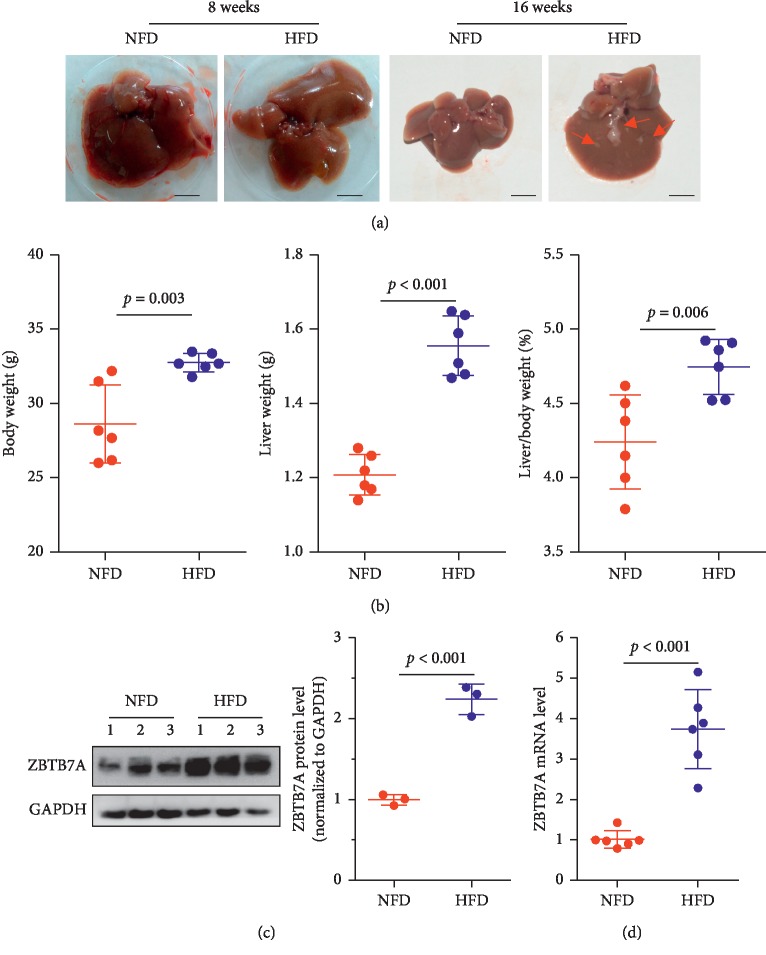
ZBTB7A was induced *in vivo*. (a) The mouse was fed with a high-fat diet for 8 weeks or 16 weeks. (b) The body weight and liver weight were collected, and the hepatic index was calculated by the liver/body weight ratio (*n* = 6). (c) Three liver tissue samples from each group were subjected to western blotting and the protein expression of ZBTB7A was examined. The right panel shows the semiquantitative statistic with Quantity One software. (d) The liver sample per group was applied in qRT-PCR to evaluate the mRNA expression of ZBTB7A (*n* = 6).

The C57BL/6 mouse was fed with a high-fat diet to construct the NAFLD model, and as displayed in Figure 1(a), with an 8-week HFD induction, the color of the liver became much lighter and was in a different amount with fine grain. With another 8-week induction with a high-fat diet, the size of the liver has become much larger and the mouse's body weight and liver weight were both increased. And, as the liver/body weight ratio is a common liver index [24], the liver index of the high-fat diet group was remarkably higher than the normal fatty diet group (Figure 1(b)). To determine the role of ZBTB7A in the progression of NAFLD, we examined the expression of ZBTB7A in the liver, and western blotting showed that HFD induced ZBTB7A protein expression (Figure 1(c)). And the mRNA level was consistent with the protein expression level (Figure 1(d)).

**Figure 2:**
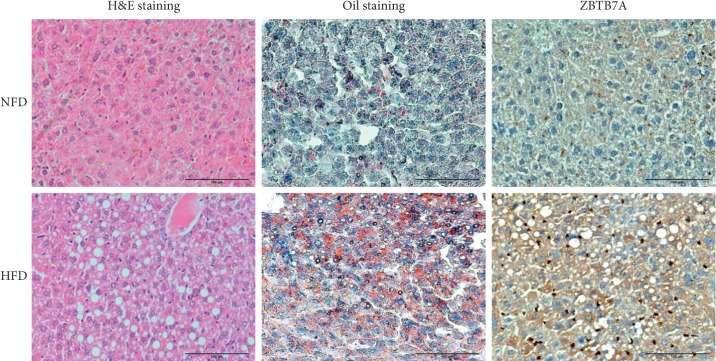
Histological evaluation of the NAFLD mouse model. H&E staining was used to evaluate the pathological features. The accumulation of lipid droplets was evaluated by Oil Red staining. And the immunohistochemistry analysis was applied to test the expression of ZBTB7A.

To further confirm the construction of the NAFLD mouse model, the liver tissues were subjected to histological analysis. As shown in Figure 2, hematoxylin and eosin (H&E) staining showed there are various vacuoles in the liver of the HFD-induced group. And Oil Red staining revealed that lipid droplets were accumulated in the liver of the HFD group. Furthermore, immunohistochemistry analysis against ZBTB7A in the liver section clearly showed that ZBTB7A expression was much enhanced with the induction of a high fatty diet. These data suggested that ZBTB7A expression was induced with a high-fat diet and might participate in the progression of NAFLD.

### 3.2. ZBTB7A Was Correlated with the Pathological Factors of NAFLD

**Figure 3:**
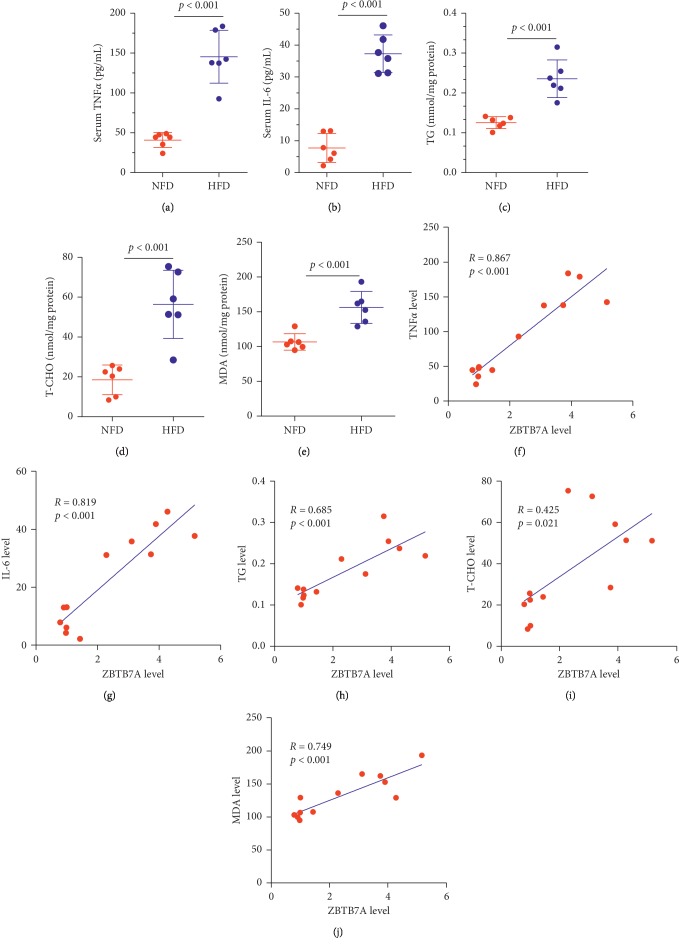
ZBTB7A was correlated with the pathological factors of NAFLD. (a) The serum TNF*α* was detected with the ELISA kit. (b) The serum IL-6 was detected with the ELISA kit. With the homogenate treatment, the liver was subjected to test the (c) TG concentration, (d) T-CHO concentration, and (e) MDA levels. (f–j) The correlation between ZBTB7A and serum TNF*α*, serum IL-6, hepatic TG, hepatic T-CHO, and hepatic MDA activity was explored with the Pearson correlation coefficient.

To fully determine the potential role of ZBTB7A in the NAFLD, the NASH mouse model was constructed with 16-week high-fat diet, and the ELISA analysis revealed that serum TNF*α* and IL-6 levels were both upregulated (Figures 3(a) and 3(b)). And the lipid metabolic molecules analysis showed that the hepatic triglycerides (TG) and hepatic total cholesterol (T-CHO) were elevated in the HFD group (Figures 3(c) and 3(d)). And the liver with the HFD-induced group has obviously higher MDA levels (Figure 3(e)). To explore the role of ZBTB7A in the NASH progress, the correlation between ZBTB7A and TNF*α*, IL-6, TG, T-CHO, and MDA was examined, and ZBTB7A showed a positive correlation with these factors (Figures 3(f)–3(j)). Thus, ZBTB7A was closely correlated with the pathological factors of NAFLD and might involve in the regulation network of NAFLD.

### 3.3. Oleic Acid-Induced ZBTB7A Expression and Lipid Accumulation *In Vitro*

**Figure 4:**
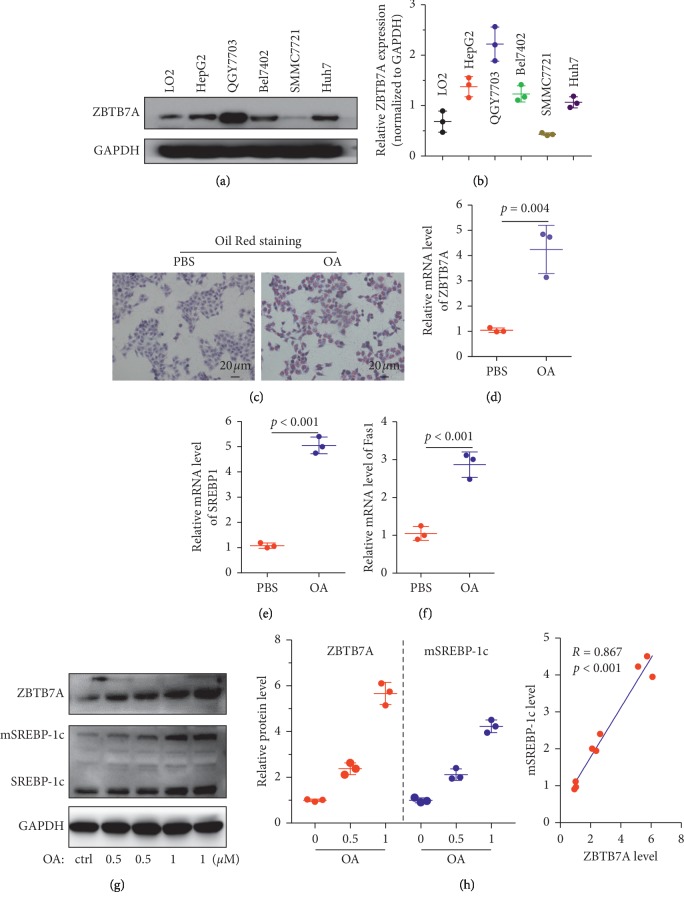
ZBTB7A was induced *in vitro*. (a) ZBTB7A expression was evaluated in LO2 normal liver cells and liver cancer cell lines. (b) ZBTB7A protein expression in several cell lines was analyzed with semiquantitative statistics with Quantity One software. (c) HepG2 cells were treated with oleic acid at the concentration of 1 *μ*mol/L, and with another culture for 24 h, the cells were stained with Oil Red reagent and costained with crystal violet to display the cell nucleus. (d–f) The HepG2 cells were treated with 1 *μ*mol/L oleic acid for 24 h, and qRT-PCR was subjected to detect the mRNA levels of ZBTB7A, SREBP1, and Fas1. (g) HepG2 cells were treated with different doses of oleic acid, and western blotting was used to reveal the protein levels of ZBTB7A and SREBP-1c. The SREBP-1c antibody could recognize the precursor and mature forms of SREBP-1c. (h) The protein expression of ZBTB7A and that of SREBP-1c were quantified with a semiquantitative statistic, and the right panel shows the Pearson correlation coefficient between ZBTB7A and mature SREBP-1c expression.

To fully understand the role of ZBTB7A in liver disease, ZBTB7A expression was identified in several cell lines, including normal liver cells and liver cancer cells.  Figure 4(a) shows that ZBTB7A protein was less expressed in normal liver LO2 cells and SMMC7721 liver cancer cells but highly expressed in HepG2, QGY7703, Bel7402, and Huh7 liver cancer cells. Furthermore, mRNA levels of ZBTB7A were examined in these cell lines, and the results of the mRNA levels were in agreement with the protein expression of ZBTB7A. Taken together, these results suggest that ZBTB7A was overexpressed in the liver cancer cells, which was consistent with that reported in the non-small-cell lung carcinoma [25], and it was contrasted with prostate cancer, melanoma, triple-negative breast cancer, and gastric cancer [26–29].

To explain the role of ZBTB7A in the lipid accumulation of hepatic, HepG2 cells were induced with oleic acid (OA), and the Oil Red staining revealed that the lipid droplets were produced in a large amount (Figure 4(c)). Furthermore, the mRNA expression level of ZBTB7A, SREBP1, and Fas1 was conducted with the qPCR assay. And the results showed that OA induced ZBTB7A expression in mRNA level, as well as the sterol regulatory element-binding transcription factor 1 isoform c (SREBP-1c) and the downstream gene, fatty acid synthase subunit beta (Fas1). Additionally, western blotting showed that the incubation with oleic acid in HepG2 cells, ZBTB7A, and mature SREBP-1c (mSREBP-1c) was increased in a dose-dependent manner, and the Pearson correlation ratio between ZBTB7A and mSREBP1c was 0.867, suggesting that ZBTB7A may be involved in the regulation network of SREBP-1c (Figures 4(g) and 4(h)).

### 3.4. ZBTB7A Increased SREBP1 Expression and Activate the Activity of NF-*κ*B

**Figure 5:**
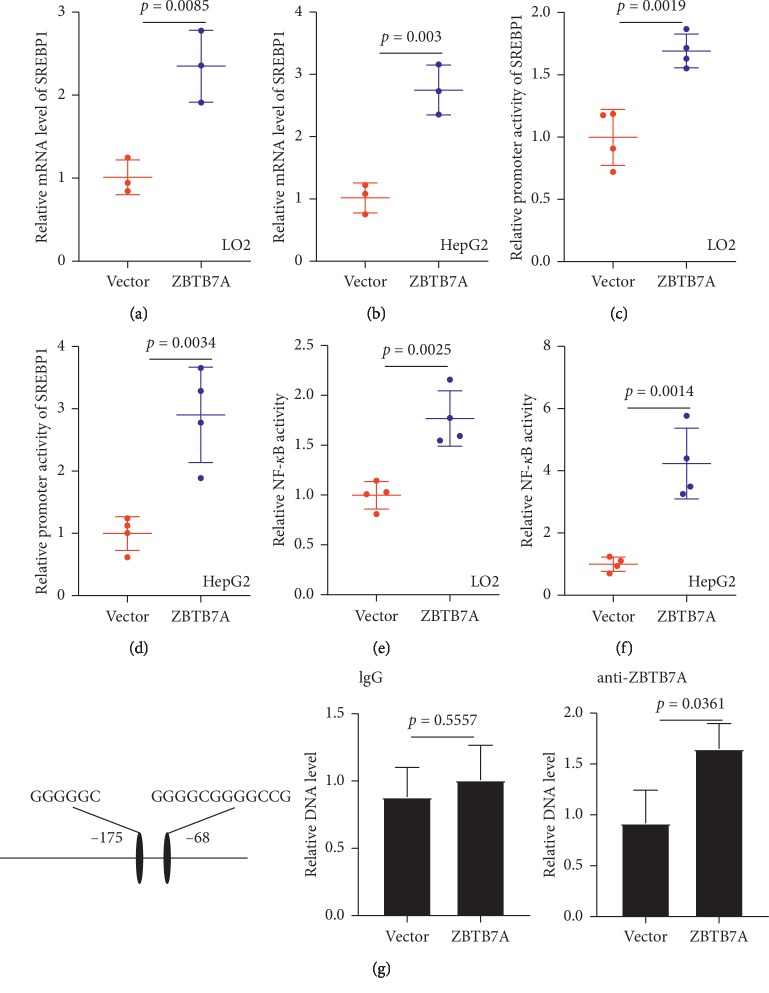
ZBTB7A increased SREBP-1c expression and activated the activity of NF-*κ*B. (a) LO2 cells and (b) HepG2 cells were transfected with ZBTB7A and vector for 24 h. The mRNA levels of ZBTB7A were examined with qRT-PCR (*n* = 3). (c) LO2 cells and (d) HepG2 cells were transfected with the pGL3-SREBP1 reporter plasmid and ZBTB7A or vector after transfected with 24 h, and luciferase reporter activity was assessed (*n* = 4). (e-f) LO2 and HepG2 cells were stably transfected with Luc-NF-*κ*B and then transfected with ZBTB7A and vector; then, the luciferase reporter activity was subjected to the luciferase reporter kit (*n* = 4). (g) The left panel shows the putative ZBTB7A binding sites within human SREBP1. And the right panel shows the ChIP assay in HepG2 cells with ZBTB7A antibody, and the IgG was used as a negative control.

As our results showed that oleic acid could induce ZBTB7A expression and promote the maturity of SREBP1. Besides, ZBTB7A was positively correlated with the maturity of SREBP1; thus, we hypothesize that ZBTB7A as a transcription factor may regulate the SREBP1. To confirm the regulation of ZBTB7A on the SREBP1, ZBTB7A was overexpressed in LO2 and HepG2 cells, and the mRNA levels of SREBP1 were remarkably enhanced (Figures 5(a) and 5(b)). With SREBP-1c as an important transcription factor, the regulation of promoter activity is a common biological process; thus, we examined the effect of ZBTB7A on the SREBP1 promoter activity, and the results revealed that ZBTB7A could increase the promoter activity of SREBP1 in LO2 and HepG2 cells, suggesting that ZBTB7A promoted the expression of SREBP1 through regulation of the promoter activity (Figures 5(c) and 5(d)).

With NASH as a higher stage of NAFLD, inflammation response is a major factor contributing to the progression of the disease [30, 31]. And our results showed that ZBTB7A was involved in the progression of NAFLD, and ZBTB7A was closely correlated with the serum TNF*α* and IL-6 levels (Figures 3(f) and 3(g)). And Essen and his colleagues have found that ZBTB7A could bind to a significant segment of promoters and enhancers of NF-*κ*B [32]; thus, we hypothesized that whether ZBTB7A regulated the inflammatory signaling. To reveal the regulation of ZBTB7A on the inflammatory response, the NF-*κ*B activity was examined, and the results disclosed that ZBTB7A could activate the activity of NF-*κ*B in LO2 and HepG2 cells (Figures 5(e) and 5(f)). To further confirm the role of ZBTB7A on the regulation of SREBP1 promoter, a previous study showed that ZBTB7A could bind to GC-rich sequence [23]. Thus, the putative binding sites were analyzed in the AliBaba2.1 portal (Figure 5(g)) to confirm whether ZBTB7A could directly bind to SREBP1 promoter, the ChIP assay was subjected, and the results showed that the anti-ZBTB7A antibody group showed increased DNA level and the IgG group as a negative control with no significant difference.

### 3.5. ZBTB7A Increased the Expression of Fatty Acid Transporter and Highly Expressed in the Hepatocytes

**Figure 6:**
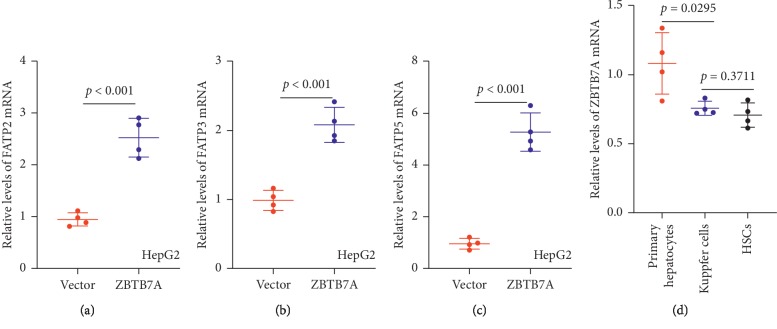
ZBTB7A increased fatty acid transporter level. (a–c) The levels of FATP2, FATP3, and FATP5 were evaluated in overexpressing ZBTB7A HepG2 and vector control cells. (d) The primary hepatocytes, Kuppfer, and HSCs (LX-2 cell line) were subjected to examine the level of ZBTB7A.

Fatty acid transporter proteins (FATPs) also play a crucial role in lipid accumulation, to study whether ZBTB7A affects the levels of FATPs. As previous reported, only FATP2, FATP3, and FATP5 were expressed in the hepatocytes; thus, in our study, HepG2 cells with overexpressing ZBTB7A and vector control cells were included to evaluate the mRNA levels of FATP2, FATP3, and FATP5. The results showed overexpression of ZBTB7A could increase the mRNA levels of FATP2, FATP3, and FATP5 (Figures 6(a)–6(c)), and these results suggest that ZBTB7A might be associated with the regulation of fatty acid transporter process. Furthermore, since Kupffer and HSC cells are involved in the regulation of NAFLD, to evaluate whether ZBTB7A mainly functions in hepatocytes, the expression of ZBTB7A in primary hepatocytes, Kupffer, and HSC cells was subjected with the qRT-PCR assay, and the results showed that ZBTB7A was also expressed in the Kupffer and HSC cells, but highly expressed in the primary hepatocytes (Figure 6(d)).

### 3.6. Knockdown of ZBTB7A Attenuated OA-Induced Lipid Droplet Accumulation

**Figure 7:**
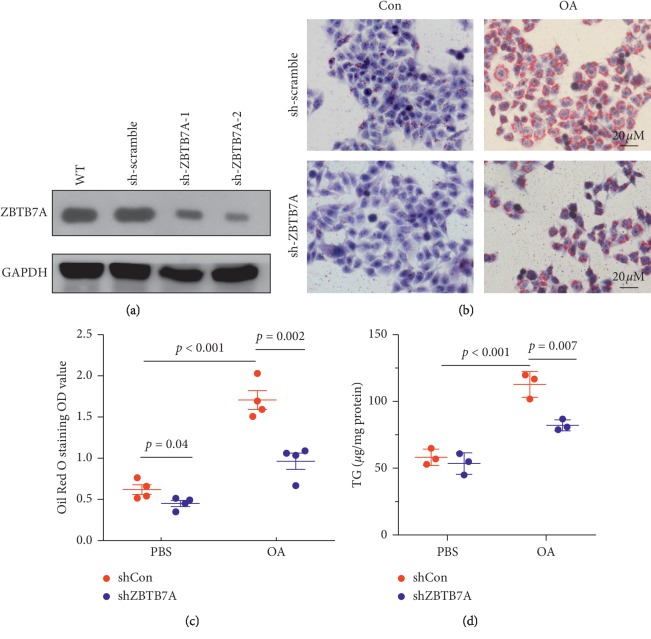
Knocking down the expression of ZBTB7A suppressed lipid accumulation induced by Oil acid in HepG2 cells. (a) Knocking down the expression with shRNA with a hairpin, HepG2 cells were transfected for 24 h, and western blotting was subjected to assess the efficiency of knockdown. (b) HepG2 cells were transfected with ZBTB7A knockdown plasmid for 24 h, and the cells were treated with oleic acid for 24 h and then subjected to the Oil Red staining and costained with crystal violet to display the cell nucleus. (c) The same procedure was applied as previously indicated, and then the cells were dissolved in ethanol, and the lipid droplets were dissolved in the solution and then subjected to microplate reader at 450 nm (*n* = 4). (d) The cells were treated as indicated, and the cell medium was subjected to the TG concentration ELISA kit, and the total protein was assessed by BCA methods (*n* = 3).

To examine whether ZBTB7A regulates the OA-induced lipid droplet accumulation, shRNA with hairpin was subjected, and the results showed that the second sequence of shRNA could significantly knock down the expression of ZBTB7A (Figure 7(a)), and the second was applicable to the subsequent experiment. As expected, knockdown of the expression of ZBTB7A in HepG2 cells could attenuate OA-induced lipid droplet accumulation. Furthermore, fewer lipid droplets were observed among the ZBTB7A knockdown cells than the scramble group without oleic acid induction (Figure 7(b)), indicating that knocking down the expression of ZBTB7A could inhibit the synthesis of lipid. Furthermore, a consistent result was observed in Figure 7(c), in which the quantity of lipid was assessed by absorption value at 450 nm. Moreover, TG concentration in the culture medium was evaluated by ELISA, and the results showed that OA-induced TG concentration and knocking down of ZBTB7A could decrease TG concentration induced by OA. However, without the induction of OA, the content of TG was no significantly different between ZBTB7A knockdown cells and the scramble cells (Figure 7(d)). These results demonstrated that ZBTB7A downregulation decreased the accumulation of lipid droplets induced by oleic acid.

### 3.7. Knockdown ZBTB7A Attenuated the Lipogenesis by Oleic Acid and Promoted the Oxidation Metabolism

**Figure 8:**
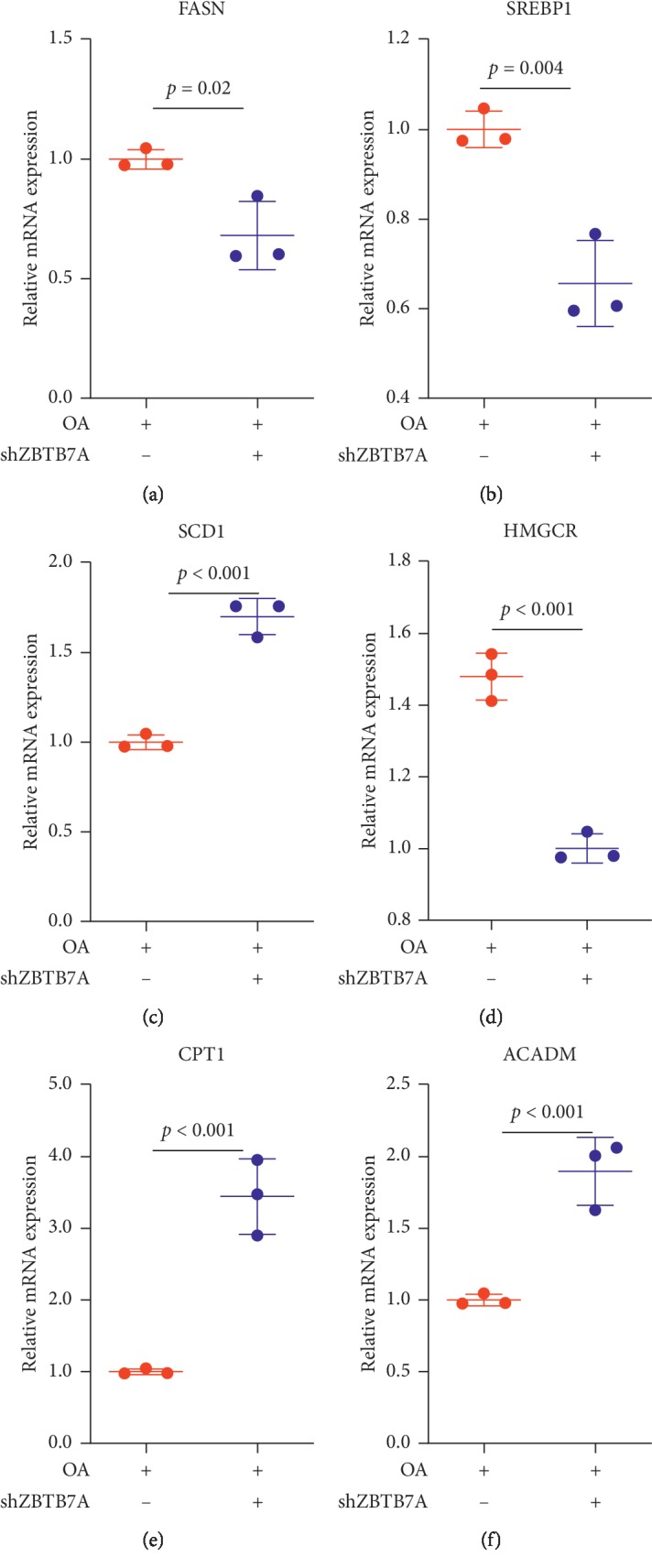
ZBTB7A was involved in the regulation of lipogenesis and fatty acid beta-oxidation. (a) HepG2 cells were stably knocked down with sh-ZBTB7A and scramble control, then treated with oleic acid for 24 h, and then subjected to test the expression of FASN. (b) SREBP1, (c) SCD1, (d) HMGCR, (e) CPT1, and (f) ACADM. The results were calculated and expressed as 2^−ΔΔCT^.

ZBTB7A played a role in the lipid accumulation and could upregulate the expression of SREBP1 through enhancement of the promoter activity. And the previous study confirmed that SREBP1 was a key factor in the regulation of lipid synthesis; also our data showed that ZBTB7A could increase the expression of Fas1 (Figure 4(f)). Then, we next attempted to explore the potential mechanism of ZBTB7A in the lipogenesis, and the results showed that knocking down the expression of ZBTB7A could inhibit the fatty acid synthase (FASN) and SREBP1 expression induced by oleic acid (Figures 8(a)–8(b)). And stearoyl-CoA desaturase (SCD1) expression was increased by knockdown of ZBTB7A (Figure 8(c)). In addition, hydroxymethylglutaryl-CoA reductase (HMGCR) as a key regulator in the cholesterol synthesis, the previous study revealed that in the lipid stress condition, HMGCR expression was in the condition of upregulation [33, 34]. Knockdown of ZBTB7A could reduce the HMGCR expression (Figure 8(d)). Considering fatty oxidation regulation was another significant aspect of the lipid accumulation. Two mitochondrial fatty acid beta-oxidation molecules, carnitine palmitoyltransferase 1 (CPT1) [35] and acyl-CoA dehydrogenase medium-chain (ACADM) [36], were elucidated, and the results revealed that CPT1 and ACADM were activated in ZBTB7A, knocking down the group, suggesting that inhibition of ZBTB7A could attenuate the lipid accumulation through the acceleration of fatty acid beta-oxidation.

## 4. Discussion

NAFLD is characterised by hepatic lipid accumulation without excess alcohol intake, and it is becoming more epidemic in the world. NAFLD includes a spectrum ranges from steatosis to NASH and has a higher risk to develop up to cirrhosis and hepatocellular carcinoma. Also, it is involved in metabolic syndromes, such as central obesity, type 2 diabetes mellitus, and hyperlipemia coronary disease. However, effective treatment for NAFLD is extremely limited except for diet and exercise because the pathogenesis has not been fully elucidated. The pathogenesis of NAFLD is reviewed as a “multihit” hypothesis that involves lipotoxicity, oxidative stress, the chronic inflammatory state, and mitochondrial dysfunction. The previous study shows that the hallmark of NAFLD is triglyceride accumulation in the cytoplasm of hepatocyte due to an imbalance between lipid acquisition and metabolism. Thus, it is an essential work to deeply understand the molecular mechanism in the occurrence and development of NAFLD.

In our study, we successfully built mice NAFLD model after 16 weeks of HFD feeding. The body weight together with liver weight and body to liver weight rate were dramatically augmented. In liver specimen hepatic steatosis, ballooning degeneration was obvious. In the HFD group, serum biomarkers such as TG, cholesterol, IL-6, TNF*α*, and MDA were also increased. Interestingly, we found that ZBTB7A was closely associated with the progression of NAFLD, and recent research also suggests ZBTB7A might be involved in the adipogenic gene expression. Then, to confirm the role of ZBTB7A in NAFLD, we evaluated the ZBTB7A expression in a mouse model. And the results showed that ZBTB7A was increased obviously at both the protein and mRNA levels. Further *in vitro* study showed that oleic acid could stimulate the expression of ZBTB7A, and knocking down ZBTB7A alleviated oleic acid-induced fat accumulation in HepG2 cells, suggesting that ZBTB7A was involved in the lipid droplet deposit. To study the potential regulation network, we examined the effect of ZBTB7A on SREBP signaling, and the results showed that ZBTB7A could activate the promoter activity of SREBP1 and significantly increase the expression of SREBP1. Additionally, a previous study revealed that ZBTB7A has the potential to bind the promoters and enhancers of NF-*κ*B, and inflammation signaling was an important mediator in the development of NAFLD. Thus, we hypothesized that ZBTB7A has closely associated with NF-*κ*B signaling, and our data confirmed the hypothesis that ZBTB7A could activate NF-*κ*B in LO2 and HepG2 cells. Furthermore, we also found that ZBTB7A accelerated lipid droplet accumulation. And ZBTB7A could promote the expression of adipogenic genes, such as FASN and SREBP1. Also, ZBTB7A could regulate the expression HMGCR, a key regulator in the synthesis of cholesterol. Based on these data, we concluded that ZBTB7A could accelerate lipid accumulation. And considering fatty oxidation, regulation was another important aspect of lipid accumulation. Two mitochondrial fatty acid beta-oxidation mediators, CPT1 and ACADM, were elucidated, and we confirmed that knockdown of ZBTB7A can inhibit the beta-oxidation through downregulation of related mediators. From lipogenesis to lipid oxidation, ZBTB7A may act as a promoter in the lipid accumulation. However, Liu, et al. demonstrated that ZBTB7A could repress glycolysis. In detail, ZBTB7A was reported to directly bind to the promoter and repress the transcription of the glucose transporter 3 (GLUT3), PFKP, and PKM [22]. And in our study, we reported that ZBTB7A could enhance the SREBP1 promoter activity, and the ChIP assay also revealed that ZBTB7A could directly bind to the promoter sequence of SREBP1 (Figure 5(g)). The diverse functions of ZBTB7A in cancer and NAFLD might be due to the commonly elevated glycolysis in cancer, and the metabolic disorder was complicated. In the future study, the transgenic mouse of ZBTB7A may be a suitable strategy for the study of ZBTB7A in NAFLD.

Taken together, in this study, we identified that ZBTB7A was overexpressed in the pressure of lipid *in vivo* and *in vitro*. The expression of ZBTB7A was positively correlated with the inflammatory factors and fatty acid synthesis factors. ZBTB7A could activate NF-*κ*B and lipogenesis-related signaling, suggesting that ZBTB7A may be a potential target for the therapy of NAFLD.

## Data Availability

The data used to support the findings of this study are available from the corresponding author upon request.
